# Songbird dynamics under the sea: acoustic interactions between humpback whales suggest song mediates male interactions

**DOI:** 10.1098/rsos.171298

**Published:** 2018-02-14

**Authors:** Danielle M. Cholewiak, Salvatore Cerchio, Jeff K. Jacobsen, Jorge Urbán-R., Christopher W. Clark

**Affiliations:** 1 Protected Species Branch, Northeast Fisheries Science Center, National Oceanic and Atmospheric Administration/National Marine Fisheries Service, Woods Hole, MA, USA; 2 Department of Neurobiology and Behavior and the Bioacoustics Research Program, Cornell Laboratory of Ornithology, Cornell University, Ithaca, NY, USA; 3 New England Aquarium, Boston, MA, USA; 4 Woods Hole Oceanographic Institution, Woods Hole, MA, USA; 5 Department of Biological Sciences, Humboldt State University, Arcata, CA, USA; 6 Programa de Investigación de Mamíferos Marinos, Departamento de Biología Marina, Universidad Autónoma de Baja California Sur, La Paz, BCS, México

**Keywords:** humpback whale song, avian song, intrasexual interactions, song matching, reproductive displays

## Abstract

The function of song has been well studied in numerous taxa and plays a role in mediating both intersexual and intrasexual interactions. Humpback whales are among few mammals who sing, but the role of sexual selection on song in this species is poorly understood. While one predominant hypothesis is that song mediates male–male interactions, the mechanism by which this may occur has never been explored. We applied metrics typically used to assess songbird interactions to examine song sequences and movement patterns of humpback whale singers. We found that males altered their song presentation in the presence of other singers; focal males increased the rate at which they switched between phrase types (*p* = 0.005), and tended to increase the overall evenness of their song presentation (*p* = 0.06) after a second male began singing. Two-singer dyads overlapped their song sequences significantly more than expected by chance. Spatial analyses revealed that change in distance between singers was related to whether both males kept singing (*p* = 0.012), with close approaches leading to song cessation. Overall, acoustic interactions resemble known mechanisms of mediating intrasexual interactions in songbirds. Future work should focus on more precisely resolving how changes in song presentation may be used in competition between singing males.

## 1. Introduction

Males of many species use vocal displays in reproductive contexts. Commonly among birds, anurans and insects, males use song (or calls) to attract females, compete with rival males, or both [1]. In an intersexual context, avian males have been shown to attract females with higher singing rates [2], larger repertoires [3], or increased song versatility [4]. While song used as a display to females may be broadcast as an omnidirectional signal, song used in intrasexual competition must be directed at specific rivals. In these cases, acoustic interaction between singing individuals should be demonstrable by measuring the response of one individual (‘receiver’) relative to the vocal behaviour of another (‘sender’). In general, males may vary song output along two dimensions to acoustically ‘point’ at specific receivers: timing (such as alternating or overlapping vocalizations with other individuals) and/or patterning (such as song matching) [5–10]. Using these mechanisms, males may use song to mediate disputes, and song characteristics such as matching [6], coordinated bout switching [7], or switching rate [8–10] may predict the escalation of aggression.

The song system of humpback whales (*Megaptera novaeangliae*) in particular has long been of interest to behavioural ecologists. The production of a long, elaborate song display is one of several alternative mating tactics used by males [11], and is arguably the most complex patterned acoustic display produced by any baleen whale. Humpback song is hierarchically structured [12,13], composed of patterns of units, which are organized into phrases. Phrases are repeated multiple times (called a ‘theme’), before the singer switches to a new phrase type. The entire sequence of themes is called a ‘song’, and males may repeat these patterns for many hours. All males within a population sing generally the same song, but song patterns evolve over time [14–17]. These changes are thought to be culturally transmitted, akin to systems in songbirds such as the village indigobird (*Vidua chalybeata*) [18,19]. Indigobirds share many mating ecology characteristics with humpback whales, for example, males continue to learn songs as adults, males are polygynous and display from leks, and there is a high degree of male–male competition (for a review, see the discussion in [16]). Different aspects of indigobird song structure and singing behaviour are thought to be driven by both inter- and intra-sexual selection [2]. Similarly, in humpback whales, complexity in song structure and dramatic divergence from sister taxa suggest the influence of strong sexual selection, and hypotheses have been proposed supporting both an intrasexual function, to mediate interactions between males [20–22], and an intersexual function, to attract females [23,24]. Female behaviour on the breeding grounds is poorly understood, and there is as yet little evidence for female choice relative to singers. However, singing males do approach and sing in association with females [25–27], though their likelihood of singing while joining a female or other groups is affected by the presence of other singers and density of whales in at least some contexts [28]. It is quite possible that song may function both to attract potential mates and to mediate interactions with other males, as is also seen in many songbird species (e.g. [29,30]).

Much humpback song literature to date has emphasized the potential use of song to mediate male–male interactions, but the acoustic mechanism by which this may actually occur has never been explored. Metrics typically used in studies of song in avian interactions (i.e. switching rates, versatility, etc.) may prove helpful. However, measuring acoustic interactions between singing humpback whales as has been traditionally done in avian systems is complicated by the following factors: (i) males sing continuously (i.e. pauses between phrases are not longer than pauses between notes; [12]), therefore timing changes are difficult to detect; (ii) all males within a population sing from the same ‘repertoire’ [14], so that all phrase types are essentially shared among individuals (with some rare exceptions), (iii) males sing with ‘eventual variety’ [13], that is, they repeat the same phrase type multiple times before switching to a new phrase type. Identifying acoustic response in eventual variety singers can be more difficult than in species that sing with immediate variety (e.g. species that sing a different phrase or song type with each repetition) [5]. Finally, (iv) humpback song is repetitive in structure such that individuals typically sing a sequence of phrase types (or themes) in a similar order [12]. Therefore, some degree of thematic overlap between songs from coincident singers is expected even in the absence of directed interactions, and this must be distinguished from intentional song overlap if males are using this feature to direct interactions with one another.

Despite these challenges, there is variation both within and between the songs of individuals [31], the function of which has not been systematically tested. While the overall timing of song components (i.e. phrases) is very stable, males may vary the number of times phrase types are repeated, and to some extent may vary the order in which themes are sung, and these variables could play a role in acoustic interactions between individuals. The goal of the current study is to evaluate the hypothesis that male humpback whales use song to mediate intrasexual interactions with other singers. If singing males are interacting with one another acoustically, then we predict that there will be measurable changes in a male's song presentation in the presence of potential rivals as compared to when he is singing alone. Accordingly, we measure three acoustic parameters: the rate of switching between phrase types (‘switching rate’), the proportional representation of all phrase types, or relative ‘evenness’ in song presentation, and finally, the level of thematic overlap between the song sequences of simultaneous singers. Song parameters are quantified over fixed periods of time, rather than over ‘song cycles’ (as is common in much humpback whale literature), as the use of the latter metric is often inappropriate for measuring behavioural response [13]. Additionally, if singers are interacting, we predict that they will not be moving randomly, but will direct their movements towards one another. Singer movement is assessed using two movement parameters: separation distances and meander ratios.

## 2. Material and methods

### 2.1. Study area and data collection

**Figure 1.**
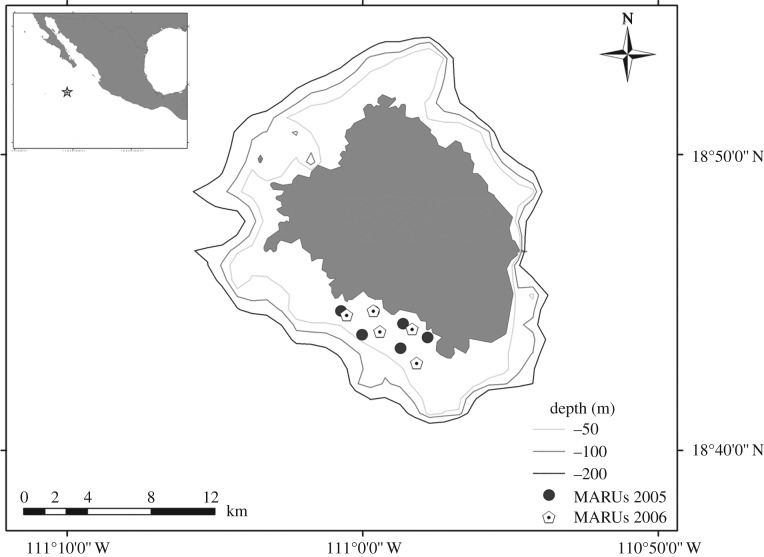
Isla Socorro, Revillagigedo Archipelago, México. Locations of the marine autonomous recording units (MARUs) for the two analysis periods are indicated by circles (2005) and hexagonal dots (2006). Note that the position for one MARU in 2005 is obscured by a MARU located at the same location in 2006.

Humpback whales were recorded off Isla Socorro, México (18°45′ N, 110°59′ W), during 2005 and 2006, using marine autonomous recording units (MARUs [32], HTI 94-SSQ hydrophone, flat (±3 dB) frequency response from 10 to 585 Hz, sensitivity with preamplifier of −168 dB re: 1 V/1 µPa, 23.5 dB gain, 12-bit A/D converter). MARUs were deployed 1.5–2 km apart in a zig-zag pattern along a 6 km stretch of coastline (figure 1); seafloor depths ranged from 35 to 100 m at sites of deployment. In 2005, six units were deployed from 2 March to 12 April, recording continuously at a sampling rate of 2 kHz. In 2006, five units were deployed from 1 March to 11 April, recording from 06.00 to 20.00 local time daily at a sampling rate of 16 kHz. After MARU retrieval, data were extracted, synchronized to ±1 ms using calibration signals played at the beginning and end of deployments, and merged to yield approximately 1000 h of 6-channel recordings (2005) and 575 h of 5-channel recordings (2006). Acoustic localization accuracy was tested using synthetic signals transmitted from a known location in the array in 2005; localization error ranged from 15 to 40 m.

### 2.2. Data review and selection for analysis

Data were reviewed visually and aurally using spectrograms (512 pt. FFT or 1024 pt. FFT, Hamming window, 50% overlap) generated in the software packages Raven [33] and XBAT [34]. Song structure was characterized at the level of phrase types [12], and singing activity was quantified by counting the number of singers present in the 10 min period at the beginning of every hour. Based on the initial review of the data, hourly samples in which one singer was detected followed by an hourly sample in which more than one singer was detected were examined further.

To select time periods for analysis of potential singer interactions, the following criterion was used: only one singer was audible across the array for at least 30 min (hereafter referred to as the ‘focal singer’), after which time a second male began singing in the area (‘second singer’). Over 40 periods were flagged and reviewed completely to determine whether the focal singer was non-intermittent (so that there would be no possibility of switching ‘focals’), and whether both singers were sufficiently close to the array to enable consistent acoustic detection.

### 2.3. Song pattern analyses

A total time window of 90 min was chosen for analysis, including up to 45 min in which the focal singer was the sole singer audible across the array (hereafter referred to as the ‘*BEFORE*’ period), and up to 45 min in which the focal plus one or more other singers were present (hereafter referred to as the ‘*DURING*’ period). Both periods were then subdivided into three 15 min time windows to enable more discrete temporal resolution. Past studies have shown that males typically cycle through all of their phrase types within 15 min or less [31], therefore, these time windows would encompass variation in song presentation and potentially capture salient responses on the part of the singing males.

Three parameters of song pattern were analysed: (i) switching rate, (ii) song evenness, and (iii) thematic overlap (or ‘song matching’). These parameters are defined below:

#### 2.3.1. Switching rate (SR)

Switching rate is defined as the number of transitions from one phrase type to another (Tn), divided by one less than the total number of all phrases (Pn):

$$\text{SR}=\frac{\text{Tn}}{\text{Pn}-1}.$$

SR was calculated for each of the 15 min time windows and averaged for the *BEFORE* period (for focal singers) and *DURING* period (for focal and second singers).

#### 2.3.2. Relative song evenness (J′)

A modified version of the Shannon index [35]:

$$J^{\prime }=-\sum_{i=1}^{k}\;(\frac{p_{i}*(\text{log }p_{i})}{\text{log }k}),$$

where *p_i_* is the proportion of observations (i.e. phrases) found in category *i* (i.e. distinct phrase type), and *k* is the number of categories (i.e. number of phrase types). The result is a quantity between 0 and 1. A score of 0 indicates very ‘low evenness’, such that some phrase types are particularly over- or under-represented due to a male singing some phrase types much more than others. Conversely, a score of 1 represents complete evenness, such that all phrase types are equally represented. Because humpback song changes over time, it was important to calculate *J*′ with respect only to the phrase types that were present in each individual's song at the time of recording. *J*′ was calculated for each of the 15 min time windows and averaged for the *BEFORE* period (focal singers) and *DURING* period (focal and second singers).

Switching rates and *J*′ were arcsine transformed and paired *t*-tests were used to test: (i) whether the focal singers changed their SR or *J*′ between *BEFORE* and *DURING* periods, and (ii) whether the focal and second singers differed significantly in the *DURING* period. Statistical analyses were performed using R [36].

Because switching rate and song evenness may be conventional signals that function differently among species [37], and we have no *a priori* information as to how these variables may function in humpback whale song, we initially conducted two-tailed tests, but report both one- and two-tailed test results in some cases.

#### 2.3.3. Phrase type overlap (e.g. ‘song matching’)

Because humpback song is continuous (i.e. pauses between phrases are not longer than pauses between notes) and cyclical, and all males generally sing the same phrase types in a similar order, it was not possible to test for song matching by traditional methods used in avian studies (e.g. [6,10]). Therefore, we chose to test for ‘matching’ between singers by quantifying how often the two singers were observed singing the same phrase type in the *DURING* period, as compared to what would be expected by chance. A Monte Carlo procedure was used to construct a distribution of how often the singers would be singing the same phrase type if their song sequences were randomly shifted in time relative to one another. This represents the ‘null’ distribution, or the amount of overlap between the males' songs that could be expected by chance, if they were not interacting acoustically. This procedure was conducted separately for each singer dyad. The steps are detailed below:

I. Quantification of the observed phrase type overlap between the song sequences of the focal and second singers in the *DURING* period. The phrase type that each male was singing was quantified every 8 s, for the length of the period. The number of samples in which the focal and second singers were singing the same phrase was used as the test statistic.
II. Monte Carlo simulations to create the ‘null’ distribution of phrase type overlap for each singer dyad. A new, random start time was chosen for the song of the second singer within the *BEFORE* period. The entire song sequence of the second singer was shifted to this new start time, preserving his sequence of phrase types. This allowed for quantification of ‘random’ overlap between the two singers. The phrase type that each male was singing was quantified every 8 s; this was used as the null test statistic. For each singer dyad, this process was performed 1000 times to generate a null distribution for phrase type overlap, or the distribution expected if the song sequences of the two males were completely independent of one another.
III. Comparison of the *observed* phrase type overlap with the *expected* distribution. The probability of the observed level of thematic overlap for each dyad was calculated from their null distribution for the overall *DURING* period.

Monte Carlo simulations for phrase type overlap analysis were performed in R. A total of 1000 Monte Carlo simulations were conducted for each dyad. Each pair was treated as an independent test, and the probability of observed phrase type overlap under each dyad's null distribution was calculated.

### 2.4. Movement pattern analysis

Singer locations were calculated from song units received on 3 or more MARUs using the correlation sum estimation method [38]. To increase the likelihood of localization accuracy, up to 10 different song units within 60 s were localized, and the median position was used as the singer's location for that minute. In most cases, the spread of possible locations was within 100 m of the median in the x and y dimensions. Singer locations were calculated up to every 2.5 min, although surfacing events and movements out of the array area occasionally decreased the ability to calculate positions at this time resolution. Interpolated tracks were generated for all singers using ISRAT [39].

To test whether the simultaneous singers were distributed non-randomly with respect to one another, three spatial parameters were quantified. These included: starting separation distance, ending separation distance, and change in distance between singers. The observed separation distances and change in distance between singers were quantified based on localization analyses described above. To test for significance, these data were compared against a null distribution for each of these variables based on measurements generated from non-interacting singers. Null distributions were generated by localizing solo singers (defined as when no other singers were audible across the array) and superimposing their tracks in time as though they had been singing simultaneously. Because in reality these males were not singing at the same time and therefore could not have been interacting, their movements with respect to one another can be considered random. Eighteen solo singing males (recorded on different days or after at least one hour during which no singing was detected) were localized as described above. Starting and ending x-y positions of each singer are included in electronic supplementary materials, S1. To generate the null distribution of starting and ending distances between singers, their locations were calculated at the beginning and end of a 45 min period, and the initial separation, ending separation, and change in distance between all possible pair-wise combinations (*n *= 153) of these singers were used to generate the null distributions.

In addition, directional travel for each singer was measured using meander ratio. Meander ratio was defined as the total distance a singer travelled divided by the straight-line distance between his initial and end position. By this measure, an individual travelling in a straight line will have a meander ratio of 1, while an individual who is milling will have a meander ratio much higher than 1. The distance travelled by each singer was calculated over sequential 5 min time bins to minimize potential errors associated with oversampling tracks. A single meander ratio was calculated over the entire 45 min *BEFORE* period for each focal singer, as well as for the focal and second singers in the *DURING* period.

All movement analyses were tested using non-parametric tests. The distribution of observed starting distances, ending distances, and change in distance between singers were tested against the null distributions using a Kolmogorov–Smirnov (K-S) test. A Mann–Whitney *U*-test was employed to determine whether change in distance between singers was related to the cessation of song by one of the individuals. A Wilcoxon-signed rank test was used to determine whether focal singers significantly changed their meander ratio between the *BEFORE* and *DURING* periods, and a Mann–Whitney *U*-test was used to test whether focal and second singers differed significantly in the distribution of their meander ratios.

## 3. Results

Based on the initial review of the data, 12 periods were identified for analysis of potential singer interactions. The duration of the *BEFORE* period for these dozen singers ranged from 35.3 to 46.1 min, with an average of 43.3 min. In eight cases, both the focal and second singer continued singing for the 45 min *DURING* analysis period. In two cases, the focal singer quit singing in the *DURING* period, after 14 min and 4 min. In another two cases, the second singer quit singing in the *DURING* period, after 23 min and 40 min.

### 3.1. Song pattern analyses

**Figure 2.**
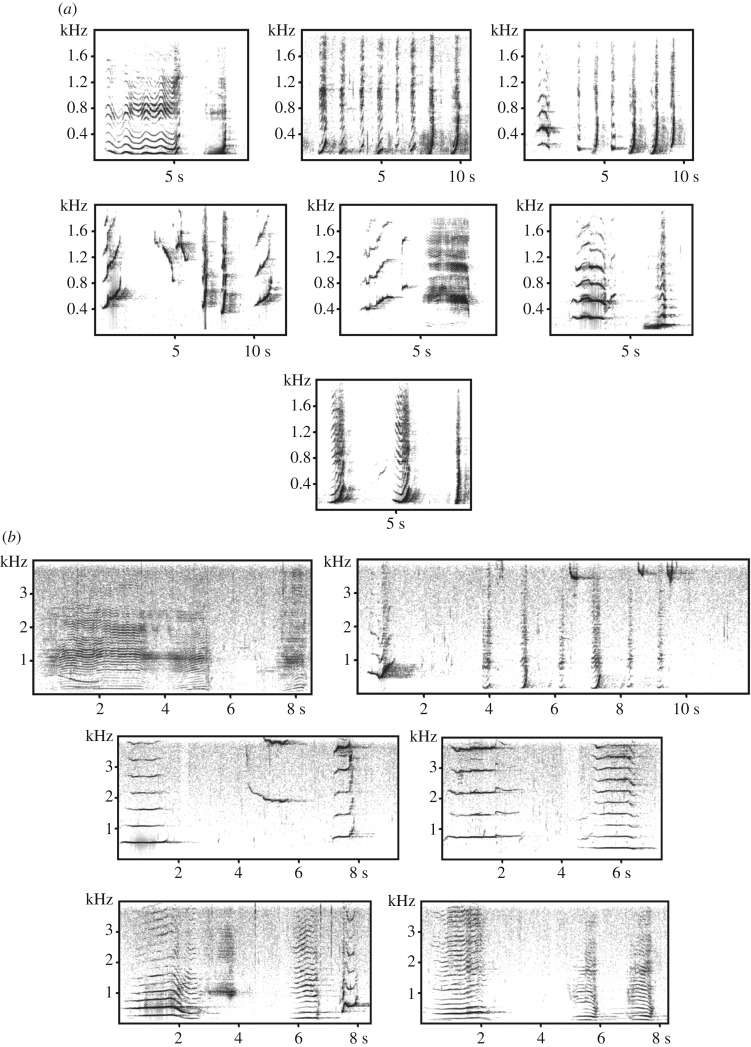
Examples of phrase types found in the population-wide song in (*a*) 2005 and (*b*) 2006. Note that the frequency axis is higher for the 2006 example due to the presence of higher-frequency units. Because humpback song evolves over time, these examples may be considered ‘snapshots’ of the phrase types at one point in time during each season.

Approximately 13 different phrase types were identified between 2005 and 2006, although no more than 10 were present in the population-wide song at any one time (figure 2). Dyad 10 was excluded from song pattern analyses as the focal male stopped singing after 4 min in the *DURING* period; the second singers from two dyads were excluded from song pattern analyses due to poor recording quality, resulting in the inability to reliably delineate every phrase. Focal males sang an average of 240 phrases in the *BEFORE* period, and 228 phrases in the *DURING* period. Focal singers significantly increased their mean switching rate from 0.11 ± 0.040 s.d. in the *BEFORE* period, to 0.14 ± 0.033 s.d. in the *DURING* period while in the presence of another singer (table 1, *n* = 11, *t*-Ratio: −3.55, *p* = 0.005, two-tailed). The mean switching rate for the second singer in the *DURING* period was 0.12 ± 0.02 s.d., which was not significantly different from that of the focal singer (*n* = 9, *t*-Ratio: 0.88, *p* = 0.4, two-tailed).

**Table 1.** Mean switching rates and relative song evenness measures for focal and second singers. Focal singers showed a significant increase in switching rate in the presence of a second singer, and a trend towards an increase in relative song evenness. Switching rates and relative song evenness were not significantly different between the focal singers and the second singers. Italics indicate results that are significant at α < 0.05.

		mean ± s.d. *BEFORE*	mean ± s.d. *DURING*	*p*-value—two-tailed test
switching rate	focal singer: *BEFORE* versus *DURING* (*n* = 11)	0.11 ± 0.035	0.14 ± 0.033	*0.005*
	focal singer versus second singer: *DURING* (*n* = 9)	0.14 ± 0.033	0.12 ± 0.02	0.4
relative evenness	focal singer: *BEFORE* versus *DURING* (*n* = 11)	0.76 ± 0.12	0.83 ± 0.11	0.06
	focal singer versus second singer: *DURING* (*n* = 9)	0.83 ± 0.11	0.83 ± 0.09	0.75

Relative song evenness (*J*′) ranged from 0.50 to 0.94 among males. On average, *J*′ for focal males in the *BEFORE* period was 0.76 ± 0.12 s.d. and increased in the *DURING* period to 0.83 ± 0.11 s.d. This change was not significant under the two-tailed *t*-test (*n* = 11, *t*-Ratio: −2.08, *p* = 0.06, two-tailed), but was significant under a one-tailed test for increase (*p* = 0.03). In the *DURING* period, *J*′ for second singers averaged 0.83 (±0.09). Focal males and second singers did not differ in their relative evenness of song presentation (*n* = 9, *t*-Ratio: 0.32, *p* = 0.755, two-tailed). Individual results for each dyad are found in electronic supplementary material, S2.

Eight pairs of singers were analysed for phrase type overlap. The proportion of overlap between the songs of singer dyads expected under a null distribution ranged from 0.13 to 0.27, whereas the observed proportion ranged from 0.10 to 0.29 (table 2). The probability of occurrence of the observed overlap in the null distribution revealed that two-singer dyads matched their song sequences significantly more than expected (*p* = 0.016 and *p* = 0.031). Interestingly, another singer dyad had a tendency to avoid matching (*p* = 0.92, or *p* = 0.08 on the left side of the distribution). The remaining five dyads showed no tendency to match or avoid matching more than expected when compared to the null distribution.

**Table 2.** The probability of observing the measured level of thematic overlap (or ‘song matching’) between two singers, based on comparison with the null distributions of overlap generated by 1000 Monte Carlo simulations for each dyad. Matching analyses were conducted for eight of the singer dyads. Italics indicate results that are significant at α < 0.05.

singer dyad	mean expected overlap	mean observed overlap	*p*-value
1	0.26	0.27	0.28
2	0.15	0.10	0.52
3	0.16	0.25	*0.02*^a^
4	0.18	0.13	0.56
5	0.13	0.13	0.51
6	0.13	0.26	*0.03*^a^
7	0.23	0.16	0.92^b^
8	0.27	0.29	0.43

^a^Two-singer dyads overlapped in their theme sequence significantly more than expected.

^b^An additional dyad showed a tendency to avoid matching.

### 3.2. Movement pattern analyses

**Figure 3.**
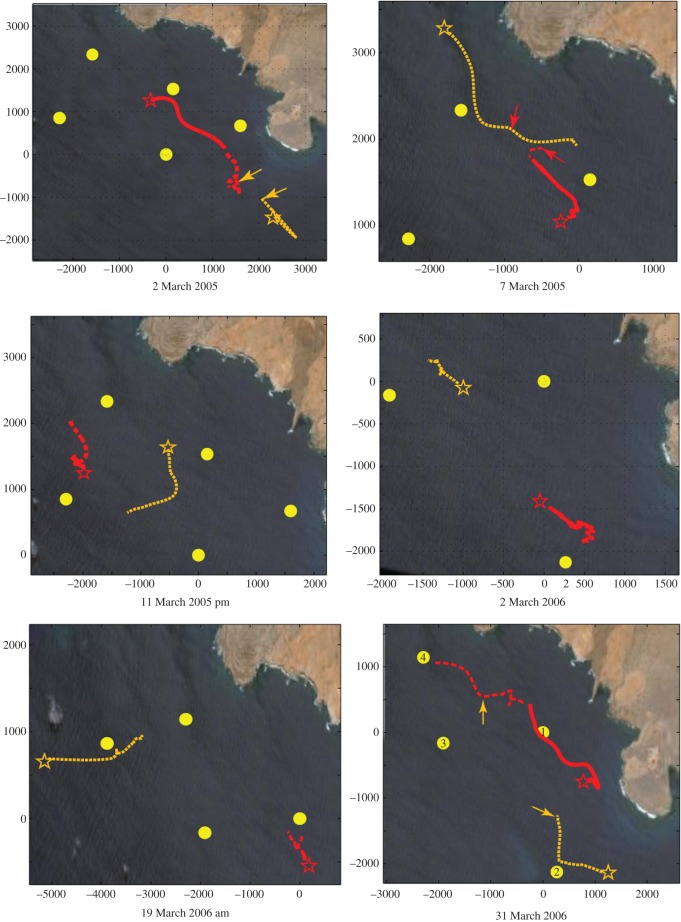
Six examples of interpolated tracks of singer movements generated from acoustic locations calculated every 2.5 min or more. Recording units are indicated by yellow circles. Focal singers are in red, the solid line indicates the singer's track in the *BEFORE* period, the dotted line indicates his track in the *DURING* period. Second singers are in orange. Stars represent the initial position for each singer. In three cases, one singer quit singing in the *DURING* period; the position of each singer at the time when one quit is indicated by the arrows, and the colour of the arrow indicates which singer quit singing. The x and y axes indicate meters from the center of the array.

Individual singers displayed a wide variety of movement patterns (figure 3). Some were essentially stationary, while others were clearly travelling during the analysis period. Several singers approached one another, while others maintained inter-individual distances.

**Figure 4.**
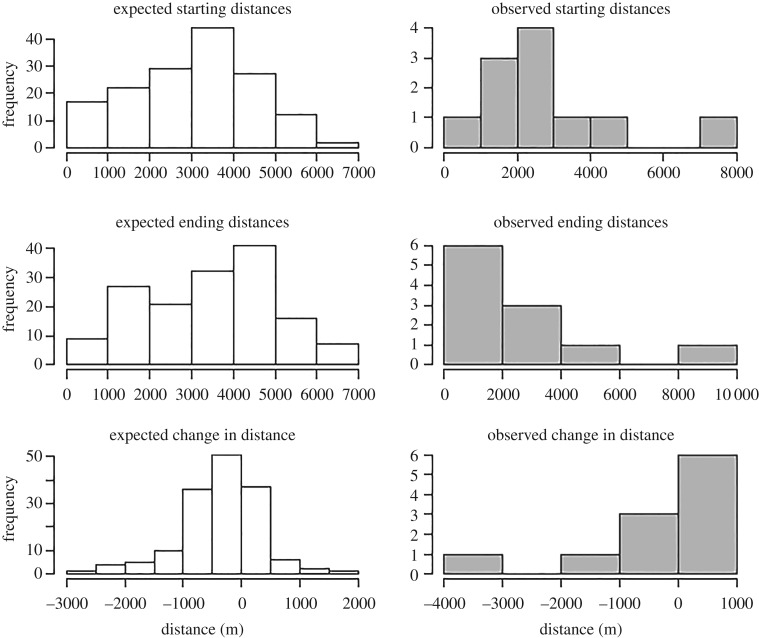
Null (‘expected’) and observed distributions of starting separation distances, ending separation distances, and change in distance between two singers. Null distributions are calculated using pairwise comparisons of locations and movements of 18 solo singers. Observed distributions are based on locations and movements of dyads of simultaneously singing males.

Based on the null distributions generated from 153 pairwise comparisons of non-interacting singers (figure 4), the mean starting separation distance between singers would be 3060 ± 1518 m if males were distributed randomly with respect to one another, and the mean ending separation distance would be 3420 ± 1590.7 m. The mean change in distance between pairs of non-interacting singers in the null distribution was 362 ± 702.5 m.

**Figure 5.**
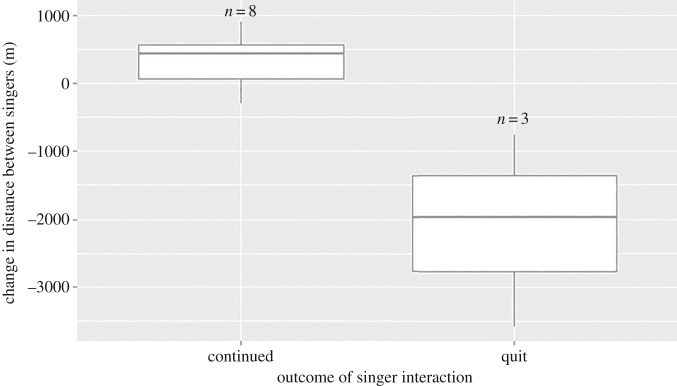
Box plot of the change in distance between the focal and second singers during the 45 min analysis period, grouped by whether both males continued singing throughout the analysis (*n* = 8), or whether one male quit singing (*n* = 3). The mean change in distance between members of dyads in which both males continued to sing was 336 ± 443.3 m, while the mean change in distance between dyad members when one singer quit singing was −2101.7 ± 1410.69 m, where negative indicates that the distance between singers decreased. The change in distance was significantly different between the two categories (Mann–Whitney: *z *= −2.18, *p* = 0.0293).

The observed distance between two simultaneous singers when the second male started singing ranged from 830 to 7500 m, which was not significantly different from that predicted by the null distribution (*n* = 11, K-S test, *p* = 0.29). However, males were significantly closer to one another at the end of the analysis period, with ending distances ranging from 540 to 8400 m (*n* = 11, K-S test, *p* = 0.015). While the change in distance between singers was not significant across the entire sample (*n* = 11, *p* = 0.089), it was significantly correlated with whether one male quit singing or both continued singing: a male was significantly more likely to quit singing if one male approached the other (*n* = 11, Mann–Whitney *U*-test, *p* = 0.012, figure 5).

Focal singers did not change the relative degree of directionality in their swimming patterns in the presence of another singer. The meander ratio for focal singers in the *BEFORE* period (2.5 ± 1.68) was similar to the *DURING* period (2.84 ± 1.53; *n* = 9, *p* = 0.426). However, in the *DURING* period, second singers were significantly more directional in their swimming patterns than the focal singers (average meander ratio: 1.49 ± 0.40, *n* = 9, *p* = 0.004). See electronic supplementary material, S3 for details for each dyad.

## 4. Discussion

### 4.1. Significance of song pattern analyses

Song parameters such as switching rate, relative evenness, and matching have been shown to be important in intrasexual interactions in a variety of species, notably songbirds. Previous studies of humpback whale song have proposed that males may use song to mediate interactions between themselves and other singers, but a mechanism by which this might be accomplished has never been investigated. This study provides a first attempt to evaluate and quantify ways in which males may use song to direct interactions with one another, applying the metrics traditionally used in the study of songbird systems.

The song pattern analyses reported here suggest that humpback males respond to the presence of another singer by increasing the rate at which they switch between phrase types as compared to when they are the sole audible singer. Correspondingly, there was a trend towards increase in the relative ‘evenness’ of song presentation in the presence of another singer, indicating that males become less repetitive and instead cycle through all of the phrase types in their song sequence more consistently than when they are alone. Finally, in two dyads, males also overlapped their song sequences more than expected by chance, similar to ‘song matching’ in songbird systems. While the sample for this study was relatively small, taken together, these results strongly suggest that singers change their acoustic behaviour in response to the presence of another singer.

Any or all three of these measures have been correlated with differing degrees of agonism between males in songbird systems (for a review, see [40]). Song type switching has been suggested to provide graded information about the motivation of the singer in agonistic contexts [7], though it is also considered a ‘conventional’ signal [41], such that it is used differently among species. In some species, such as the banded wren (*Thryophilus pleurostictus*) [42], males appear to sing in a more repetitive, less versatile mode when alone, and switch to more versatile singing in the presence of other singing males. In that study, the authors suggest that this allows potentially rival males to assess one another more fully, before deciding whether to escalate an interaction. Further work on that system has demonstrated that these variables are employed in singing interactions to provide graded information about the singer's motivation and are used in boundary negotiations [43]. Furthermore, song type matching allows males to unambiguously ‘direct’ their singing behaviour to another male, and has been suggested to be a signal of low-level escalation in some species [40].

The fact that humpback whales modify their song presentation in ways similar to songbirds, at least in some situations, suggests a homologous process of sexual selection. Thus, as has previously been emphasized [11,20], the same evolutionary principles guiding studies of sexual selection and mating behaviour in terrestrial taxa can be applied to cetaceans as well. The difference in song presentation by the focal singer before and after the second male started to sing demonstrates that the focal singer changed his behaviour in response to the presence of a second male, potentially as a competitive male may treat an intruder. The fact that two dyads overlapped their themes significantly more than expected at random further supports this interpretation. Both males may be displaying a mode of singing that is indicative of competition (with high switching and high evenness, showing off the extent of their repertoire) as opposed to a mode more suited towards mate attraction (which the focal male would have been in before the onset of the second male's singing).

Smith [44] conducted a study on the behaviour of singing males while on migration, comparing the song patterns of lone singers to those of singers escorting mothers with calves. In this context, he reported that males reduced phrase repetitions in the presence of females as compared to lone singers: this could translate into a higher switching rate, as we found with male–male interactions. Therefore, it seems plausible that males use the same metrics in the context of both inter- and intra-sexual signalling (see below for further discussion).

### 4.2. Significance of the movement analyses

A predominant hypothesis within the humpback literature suggests that singing males are distributed throughout their display range and that song may function to enable singers to maintain spacing between one another [21]. Our results provide evidence contrary to this hypothesis. Although the mean distance between singers was not closer than randomly expected at the initiation of an interaction, the overall results suggest that males do approach one another more than expected, and several singers approached one another to distances of less than 1 km. Likewise, singing males were also found to approach experimental playbacks in Hawai'i when presented with similar songs [45], and approached or were approached by other singing males in a study of singer behaviour in a migratory region off Australia [27].

In our study, the ‘close approaches’ (i.e. less than 1 km) were correlated with the cessation of singing by one of the two individuals, whereas males who did not approach to such a close distance continued to sing. These results could be interpreted as the competitive displacement of one singer by another. We cannot determine what happened after one male ceased to sing, and thus whether close approaches between singers ever result in direct aggression is unknown. Based on a study of interactions between singers and other groups during migration, Smith *et al.* [27] suggested males may approach singers to prospect for females. However, this does not necessarily explain why a male would cease singing upon a close approach by another individual, unless singers were with females and therefore changed behaviour when interrupted by another male.

In either case, this pattern may be akin to that observed on terrestrial lek breeding grounds, where displaying males are interrupted by competitors. The rate at which these interruptions occur, and the degree to which they lead to aggressive interactions, vary among breeding systems. However, theory predicts that because disruption of male display is disadvantageous to both males and females, selection will act to minimize disruption through the evolution of male dominance hierarchies and male spacing [46]. The humpback whale mating system has been likened to a lek [11,47], and the spatial interactions observed in the current study may also fall into the category of dominance sorting.

It has also been suggested in the literature that humpback males may use song for ‘coalition-building’ [22], based on observations of other individuals, primarily males, interacting with singers. However, the function of these joining events is unclear, and the duration of the interactions is invariably short. It is important to note that many of these events involved a non-singing individual (or occasionally multiple individuals) approaching a singing male, who almost always stopped singing during the interaction [22]. The pattern of those interactions may also fall into the category of intrasexual display disruption that is observed on leks [46], though this was not considered as the hypothesis. Our data suggest that singers may be interacting with one another acoustically prior to, or without coming so close so as to be within visual contact with one another. Therefore, the interactions between simultaneous singers as presented in this study most likely represent a different category of interaction from those presented by Darling *et al*. [22].

Focal and second singers also differed significantly in their overall pattern of movement. In general, the movement patterns of focal singers were less directional, and in some cases similar to milling or stationary behaviour, while the second male to begin singing in the same area exhibited more directional movement. While the biological significance of this difference in movement patterns is not clear, it does suggest that the movements of singing males do appear to be influenced by the presence of other singers.

### 4.3. How are acoustic interactions and movement patterns related?

The results of both the song pattern and the movement analyses suggest that some singers are interacting directly via song, and some singers are interacting directly via close approaches. The fact that not all singers interact with one another suggests that multiple factors are involved in a singer's decision to acoustically interact with or directly approach another singer. These factors may include familiarity between individuals, assessment of competitive ability, or motivation to continue singing within a particular region of the breeding ground. This is consistent with what is seen in terrestrial taxa, where familiarity with an individual (e.g. the ‘neighbour/stranger’ paradigm often cited in avian literature), dominance status, or motivation may affect interactions between competing males. In chickadees, for example, individuals during playback experiments responded most aggressively when their songs were frequency-matched by intruders, with more agitated responses and closer distances of approach. Differences in response between males were explained in part by differences in male rank [48]. However, in a study of natural interactions between singing males on their established territories, higher levels of frequency matching were associated with higher levels of song overlap (an indication of aggression), but did not predict subsequent approaches during escalated contests [49]. Together, these studies highlight the importance of context in interpreting individual behaviour.

The next logical question is whether the ‘close approaches’ between humpback singers are mediated by the preceding acoustic display of one or both males. If males are using song characteristics to determine whether to maintain spacing, or investigate or disrupt another singer, this could provide a mechanism by which intrasexual selection could shape song characteristics in humpback whales. To evaluate this hypothesis, it is necessary to test whether phrase type overlap (‘song matching’), switching rate, or some other song parameter is correlated with the separation distance between two singers. The current sample is not large enough to allow robust evaluation of this hypothesis, but a more extensive dataset may enable us to determine which acoustic features are important for mediating these types of interactions between singers.

### 4.4. Does humpback song function as both an intrasexual and intersexual signal?

In many avian species, song has been shown to serve both an intersexual as well as intrasexual function within the breeding system. The use of different song types or changes in song presentation style may be used to direct attention to either males or females under different circumstances (e.g. [50,51]). In other cases, the same song features may be used for both female attraction and male competition, potentially leading to conflicting selective pressures [52]. Furthermore, both males and females eavesdrop on acoustic interactions and may use information obtained from those interactions to make subsequent decisions about interacting with the singers [53]. Male ornaments (such as complex song) may evolve under primarily intersexual or intrasexual selective pressure, yet be used by both sexes for assessing competitors or mates.

The current study provides both a mechanism by which humpback males may use song to interact with one another, and evidence that they do so, in at least some cases. However, this does not in any way preclude song from being used for mate attraction as well, and we would argue that the complexity and evolving nature of song, as well as the lek-like mating system, suggest that female mate choice may still be the driving selective force in the humpback song system. Although there are as-yet few data regarding female mate choice in humpback whales, Smith [44] found that singing males associated significantly more frequently and for longer periods with mother–calf pairs than with other types of groups, suggesting a role for song in mediating intersexual interactions [27]. Furthermore, our results, when taken together with the results from Smith's [44] study, suggest that similar mechanisms are employed to mediate both inter- and intra-sexual interactions. In both cases, lone males sing more repetitive songs than in the presence of other individuals, suggesting the influence of sexual selection on repertoire use in the presence of other singers or females.

## 5. Conclusion

It is clear that there are many questions still unanswered with respect to the humpback song system. Historically, it has been difficult to study the acoustic behaviour of multiple individuals relative to one another. In terrestrial taxa (e.g. songbirds or frogs), where males may be reliably located and observed simultaneously, large sample sizes of male displays may be obtained, allowing the discrimination of subtle behavioural interactions. However, the tools to simultaneously track and analyse the behaviour of multiple individuals underwater within a cetacean breeding system have not been readily available until relatively recently, and despite these advances, it is still challenging to obtain large samples. Given the small sample sizes typical of cetacean behavioural studies, it is therefore all the more important to examine the results in the context of better-studied systems, and draw from the insights acquired from those systems for interpretation.

Although the sample of individuals examined in this study was relatively small, we provide evidence that singing humpback males interact with one another, similarly to that seen in terrestrial taxa. By drawing from the wide body of theory developed for terrestrial species, this study presents an attempt to analyse humpback song using metrics frequently employed in the analysis of songbird interactions. We hope that the metrics employed in this study may enable improved understandings of the dynamics of humpback singing behaviour and provide avenues for further exploration.

## Supplementary Material

Cholewiak_etal_Solo_Start_End_Positions

## Supplementary Material

Cholewiak_etal_RSOS171298_S2_SongAnalyses_Summary

## Supplementary Material

Cholewiak_etal_RSOS171298_S3_SUMMARY_Singer_Movement_Data

## Data accessibility

The datasets supporting this article have been uploaded as part of the electronic supplementary material.
